# The New Arctic Expedition

**Published:** 1860-04

**Authors:** 


*The New Arctic Expedition.*—Dr. I. I. Hayes, who was attached to Dr. Kane's expedition in the capacity of surgeon, and who has had large experience of Arctic travel, is now organizing another Arctic voyage of discovery. Taking the open Polar sea discovered by Morton as a fixed and constant fact, he recognizes, as the only barrier to shut us out from the mysteries of the Pole, the belt of ice which floats around those undiscovered regions. Once penetrate this, and an open sea will receive the adventurous voyager, who can then unveil all the wonders which have been slumbering in these silent wastes since the creation. What will he find? Do the shores of that vast ocean swarm with birds? Does that frosty air echo to the rustle of innumerable pinions, or does it vibrate only to the sound of the fall of icebergs, and the crackle of the darting columns of the Aurora Borealis? Are its green depths stirred by the rush of fishes and great whales, and do their billows laugh and sparkle in the sun, or is all silent in the abyss, and is the surface locked in fetters of eternal frost? Are there human beings on any of the lands within that mysterious circle, and if so, who, how came they there, and what are their relations to the race?

How will the magnetic needle behave over that silent centre of the whirling earth? What phenomena will the Aurora Borealis display in its distant and unknown home? What becomes of the Gulf-Stream, that wonderful river rushing through the ocean, when it has swept beyond the rocky capes of Norway? Are those regions scourged with storms, or is there an eternal quiet brooding over the axis?

Who can answer these questions? The wide belt of ice, with its frowning battlements of icebergs, warns us back, and guards well the mysteries entrusted to its care. But Dr. Hayes thinks he can burst through the barrier, and bring us news from the inner circle. We wish him God-speed in his enterprise—a successful voyage and a safe return.

The prospect of the speedy organization of the expedition is good. The minimum amount named by Dr. Hayes is nearly subscribed, but we hope to see more liberal contributions. It is a matter of great interest, and should command the attention of all who profess any concern for the advancement of science.

